# Extracorporeal Membrane Oxygenation to Facilitate Chemotherapy—Moving the Goalposts!

**DOI:** 10.7759/cureus.29576

**Published:** 2022-09-25

**Authors:** Emad A Chishti, Thomas Marsden, Aaron Harris, Zhonglin Hao, Suresh Keshavamurthy

**Affiliations:** 1 Division of Cardiothoracic Surgery, University of Kentucky College of Medicine, Lexington, USA; 2 Division of Medical Oncology, Markey Cancer Center, University of Kentucky College of Medicine, Lexington, USA

**Keywords:** cardiorespiratory failure, testicular cancer, veno-arterial-venous extracorporeal membrane oxygenation, chemotherapy, extracorporeal membrane oxygenation

## Abstract

Extracorporeal membrane oxygenation (ECMO) is a lifesaving intervention in critically ill patients with cardiorespiratory failure. ECMO in patients with cancer is generally contraindicated not only to conserve precious resources and properly direct use but also due to a multitude of associated physiological derangements in these subsets of patients. ECMO in patients with disseminated cancer is an automatic rule-out except for anecdotal reports. Despite this, select patients with metastatic chemotherapy-sensitive cancer may benefit from ECMO as a bridge to therapy. In this report, we describe the use of veno-arterial-venous ECMO (VAV-ECMO) as a bridge to facilitate chemotherapy in a patient with cardiorespiratory failure secondary to a chemotherapy-sensitive metastatic non-seminomatous germ cell tumor.

## Introduction

Extracorporeal membrane oxygenation (ECMO) is a well-described life-support measure used in critically ill patients with a variety of conditions causing cardiopulmonary failure. In recent years, continued innovation and advancement in the ECMO domain have extended its use beyond traditional indications, allowing it to support patients with uncommon problems that are potentially reversible [1]. Use of ECMO in critically ill patients with cancer is a contentious issue given the immunocompromised state, high risk for bleeding secondary to thrombocytopenia or other hematological derangements, risk for sepsis secondary to neutropenia, and reduced life expectancy [2]. Despite this, there have been multiple reports on the use of both veno-venous and veno-arterial ECMO to facilitate chemotherapy in the management of both adult and pediatric patients with various malignancies causing cardiopulmonary compromise [3-7]. Veno-arterial-venous ECMO (VAV-ECMO) also has the potential to not only provide cardio-respiratory support but also facilitate chemotherapy in patients with metastatic malignancy.

## Case presentation

**Figure 1:**
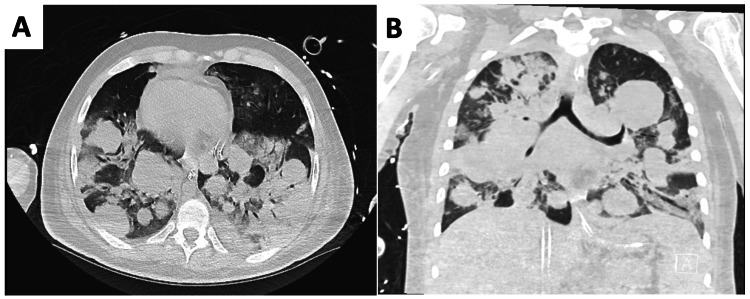
Axial (A) and coronal (B) chest CT images without contrast demonstrating extent of metastatic pulmonary disease from primary testicular non-seminomatous germ cell tumor. Both images show extensive solid nodules and masses throughout the lungs.

A 26-year-young male with fragile X syndrome initially presented to an outside hospital with testicular discomfort and shortness of breath. At this time, evaluation revealed a right testicular mass with heavy metastatic burden to the lungs, mediastinum, liver, and kidneys (Figure 1). The patient subsequently underwent a right orchiectomy and remained intubated and sedated post-operatively due to increasing oxygen requirements and eventual hypoxic respiratory failure. Biopsy results confirmed a testicular germ cell tumor, specifically embryonal carcinoma. Serum tumor markers (b-HCG and AFP) were also elevated. The tumor was staged as IIIC (TxNxM1b) with risk group classification of poor risk (an estimated 50% are cured after treatment with either four cycles of bleomycin, etoposide, and cisplatin (BEP), or four cycles of etoposide, ifosfamide and cisplatin (VIP). Given pathology findings, the oncology service at outside hospital initiated a chemotherapy regimen consisting of ifosfamide, etoposide, and cisplatin. Due to continued worsening of the patient’s respiratory status even on maximum ventilator settings, he was transferred to our institution, a tertiary care center, for further multidisciplinary management. At this time, and upon discussion with our hematology-oncology team regarding the pathology findings, it was believed that the patient’s primary testicular cancer would be responsive to chemotherapy and that he would benefit from initiation of veno-venous extracorporeal membrane oxygenation as a bridge to completion of chemotherapy. 

**Figure 2:**
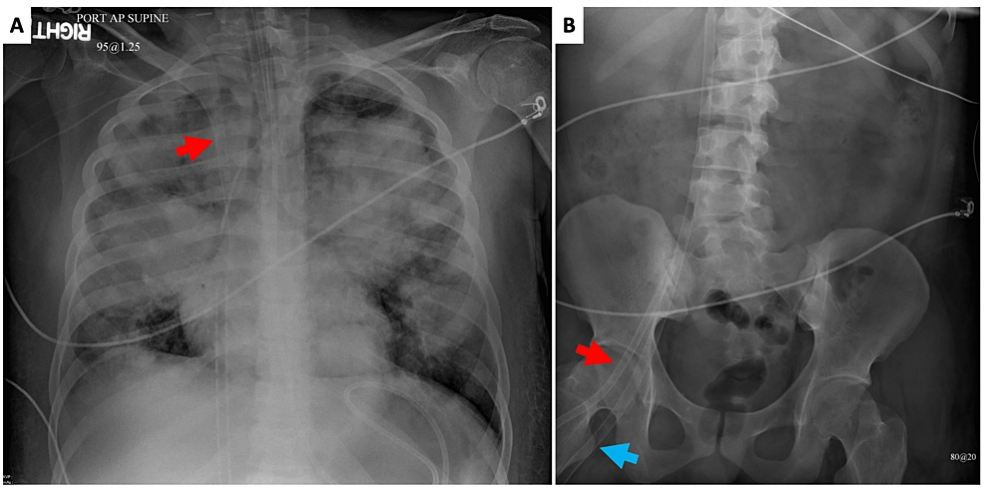
Chest (A) and abdominal (B) radiographs showing veno-arterial-venous ECMO configuration. Figure A: red arrow = 16-French right internal jugular return venous limb of the VAV-ECMO configuration. Figure B: red arrow = 17-French right femoral arterial cannula, blue arrow = 24-French right femoral venous drainage cannula. ECMO = extracorporeal membrane oxygenation; VAV-ECMO = veno-arterial-venous extracorporeal membrane oxygenation

On arrival, the patient was sedated and intubated on pressure support ventilation (100% O2, PEEP 16cmH2O) with temperature 102.2 °F, heart rate 144, and blood pressure 101/56 mmHg. The patient was maintained on vasopressor support consisting of epinephrine (0.05 mcg/kg/min), norepinephrine (0.02 mcg/kg/min), and vasopressin (0.03 units/min). Bedside transthoracic echocardiogram (TTE) showed hyperdynamic left ventricular function with normal systolic function (estimated ejection fraction 52%) and moderate-severe right ventricular dilation with moderately reduced systolic function. Significant lab values at the time included K+ 6.3, Cr 1.51, BUN 29, WBC 15.69, Hb 9.7, Hct 29.1, AST 837, and ALT 482. Arterial blood gas results were pH 7.2, pCO2 58, PO2 61, HCO3 23, and lactate 7.8. The initial plan prior to transfer was to perform veno-venous ECMO which was modified and, instead, veno-arterial ECMO (VA-ECMO) configuration was performed given the patient’s cardiopulmonary compromise. An additional venous return line was added to mitigate possible North-South syndrome with a peripheral VA-ECMO setting. We also wanted to potentiate chemotherapy response and augment flow through the pulmonary arteries to enable first-pass effect of the chemotherapy to help with early resolution of the metastases. This was the additional rationale in adding the ‘V’ limb to the VA-ECMO thereby making it a VAV-ECMO configuration. A 16-French right internal jugular venous cannula, a 24-French right femoral venous cannula, a 17-French right femoral arterial cannula, and a 6-French right superficial femoral artery distal perfusion cannula were placed (Figure 2). 

Chemotherapy regimen of ifosfamide 1200mg/m2, etoposide 50mg/m2, and cisplatin 20mg/m2 was started. A five-day cycle of these medications to be repeated every three weeks was planned. The patient had previously received day one and day two of his five-day cycle at outside hospital, thus day three of these medications was started one day following initiation of VAV-ECMO with day four beginning two days after initiation of VAV-ECMO. Prior to initiating day five of chemotherapy, the patient developed hematuria, therefore ifosfamide was held; however, the patient did complete his day five doses of etoposide and cisplatin. The following day after completing his cycle, the patient was found to be neutropenic with an absolute neutrophil count of 0.11 k/uL and thrombocytopenic with platelet count of 40 k/uL. Filgrastim therapy was initiated, and two units of platelets and one unit of packed red blood cells were transfused. On VAV-ECMO day seven, the configuration was modified to VA-ECMO due to hemoptysis. For anticoagulation, the patient was initially maintained on IV bivalirudin prophylaxis dosed at 0.08 mg/kg/hr. Our institution has moved away from the use of heparin to reduce the frequency of heparin-induced thrombocytopenia (HIT). Bivalirudin was ultimately discontinued due to coagulopathy- and chemotherapy-induced thrombocytopenia. 

The patient eventually developed acute kidney injury (AKI) with creatinine elevated to 1.99 along with volume overload and multiple electrolyte abnormalities, prompting initiation of continuous renal replacement therapy. The patient developed pancytopenia and septic shock while continuing to require multiple vasopressors. Ultimately, the decision was made to withdraw life-sustaining treatment and the patient expired soon thereafter on hospitalization day 11. 

## Discussion

Respiratory failure is among the most common reasons for ICU admissions in patients with cancer [8]. Malignancy has historically been considered a contraindication to the use of ECMO for cardiopulmonary support in these patients due to the high risk of bleeding and infection associated with the underlying pathophysiology of cancer and/or adverse effects of chemotherapy. In fact, multiple large studies have shown that both pediatric and adult patients with cancer have significantly higher mortality rates and poor survival compared to those without [9-10]. However, selective and judicious use of ECMO in specific patient populations can improve survival. Both VA-ECMO and veno-venous ECMO have been successfully described as a bridge to chemotherapy. The use of ECMO as a bridge to chemotherapy has been reported in children with hematologic malignancies [11], both children and adults with other chemotherapy-sensitive mediastinal malignancies [3-7, 12], and even a pregnant woman at full term with mediastinal lymphoma, cardiogenic shock, and superior vena cava (SVC) syndrome [13]. 

This case report describes the use of ECMO in a patient with cardiopulmonary failure secondary to metastatic testicular cancer. In this case, ECMO also uniquely serves as an adjunctive therapy to prolong survival in a critically ill patient with cancer allowing for continuation of induction chemotherapy. Although the patient ultimately expired due to widespread metastatic disease, the technical use of VAV-ECMO to allow for continued administration of chemotherapy was accomplished. After initiation of VAV-ECMO, the patient was able to receive his third and fourth dose of ifosfamide and his third, fourth, and fifth dose of both etoposide and cisplatin. While others have reported success in using both veno-venous and VA-ECMO as a bridge to chemotherapy, this report is the first, to our knowledge, to describe the attempted use of VAV-ECMO for this purpose. Tables listing common indications for ECMO and purported benefits of VAV-ECMO are provided below (Tables 1, 2). 

**Table 1:** Common indications for ECMO ECMO = Extracorporeal membrane oxygenation; ARDS = acute respiratory distress syndrome

ECMO Indication	Comments
Respiratory failure	Veno-venous ECMO can be: internal jugular / femoral, femoral/femoral/dual site, single-dual lumen cannula
Cardiogenic shock	Veno-arterial ECMO can be: peripheral (femoral artery and vein) or central (aorta / right atrium or other combinations of axillary artery / femoral vein, etc)
ARDS	Veno-arterial-venous ECMO with additional venous limb from the internal jugular to mitigate cerebral / upper body hypoxia

**Table 2:** Purported benefits of veno-arterial-venous ECMO VA-ECMO = veno-arterial extracorporeal membrane oxygenation; ECMO = extracorporeal membrane oxygenation

Purported benefits of veno-arterial-venous ECMO
Mitigate North-South syndrome with a peripheral VA-ECMO setting
Help with weaning patients on VA-ECMO with pulmonary compromise after cardiac recovery – allows for easy transition to veno-venous ECMO
During lung transplantation on peripheral VA-ECMO when hypoxia / hypercapnia needs to be resolved
To help patients with severe fibrosis / idiopathic pulmonary fibrosis as a bridge to lung transplant to maintain adequate oxygenation and CO_2_ clearance while enabling physical therapy & ambulation
To help with drug delivery, specifically chemotherapy, in the treatment of chemo-sensitive tumors of primary lung malignancy or metastatic disease in selected patients

Although our patient unfortunately expired, we would like to emphasize the following points of this case to help those intending to take care of similar patients. First, limiting anticoagulation in patients on VA-ECMO, especially those with cancer due to increased risk of thrombosis, should be considered. A study of patients on VA-ECMO showed that absence of anticoagulation was not associated with higher mortality, pump failure, or thrombotic complications [14]. We suggest that if routine systemic anticoagulation is not given, providers should monitor platelet count and other hematologic markers closely in these patients. In patients with cancer and respiratory failure, veno-venous ECMO is a reasonable management strategy and should be initiated early in the disease course, if possible, ideally prior to the onset of circulatory failure. In our case, given the burden of metastatic pulmonary disease and the patient’s late presentation with both respiratory and circulatory failure, this was not an option. As such, the decision was made to initiate VAV-ECMO. When initiating VA-ECMO in patients with chemotherapy-sensitive cancer affecting the lungs, an additional venous limb should be considered to prevent occurrence of North-South syndrome by using a peripheral VA-ECMO setting, and to also theoretically enhance regional delivery of chemotherapy, which we describe further below. 

Regional delivery of chemotherapy is a well-described approach for the treatment of locally advanced or metastatic malignancies. For patients with pulmonary metastatic disease, isolated lung perfusion (ILP) can be used to achieve regional delivery of chemotherapy. ILP is a surgical technique involving delivery of high-dose chemotherapy to the lungs while minimizing systemic exposure by delivery through the pulmonary artery with subsequent diversion of venous effluent to minimize systemic toxicity [15]. Müller and Guadagni previously described the use of isolated thoracic perfusion in addition to systemic chemotherapy in 18 patients with relapsed non-small cell lung cancer; results showed encouraging survival outcomes, minimal or transient side effects, and no treatment-related death [16]. Hengst et al. have also reported on the use of ILP in patients with various primary tumors and lung metastases treated with melphalan; early long-term survival data from this phase one clinical trial showed promising results [17]. Given these studies and other reports on the use of ILP, we hypothesized that using an additional venous limb in our patient, as described above, would prove useful in augmenting blood flow to the lungs to enhance delivery of chemotherapy. Bronchial delivery of chemotherapy is, perhaps, ideal, but cumbersome and technically difficult. On the other hand, pulmonary artery delivery is easier and enables the chemotherapeutic agents to have a first-pass effect through the lungs. While VA-ECMO diverts blood away from the lungs, the venous limb enables blood to circulate through the lungs and thereby improve delivery of chemotherapy. This is perhaps one way of using the dual blood supply of the lungs to our advantage. 

Given that ECMO is an expensive, relatively scarce life-saving measure, one could argue against the use of ECMO in the case of our patient. Malignancy has historically been considered a contraindication to the use of ECMO. The most recent Extracorporeal Life Support Organization (ELSO) 2021 Interim Guidelines for VA-ECMO in adult cardiac patients lists “poor life-expectancy" (which includes malignant tumors) as a contraindication to use [18]. Similarly, the ELSO 2021 Interim Guidelines for Veno-venous ECMO in adult patients lists “anticipated nonrecovery without a plan for viable decannulation” as the only absolute contraindication and lists “immunosuppression” as a relative contraindication [19]. Despite this, both guidelines acknowledge that ECMO, in select patient populations, may be a beneficial life-sustaining therapy despite the provided contraindications. As previously stated, there are multiple reports on the successful use of ECMO in a variety of different patient populations with hematologic and solid tumor cancers [3-7, 9-13]. As such, we believe that the use of ECMO was justified in our patient given a supportive, multidisciplinary team of surgical and medical experts who agreed upon the treatment plan, as well as patient-specific factors such as age and chemo-sensitive nature of primary tumor. Our hope was that initiating ECMO would ultimately preserve the patient’s respiratory status while chemotherapy could be delivered to decrease the burden of metastatic disease. However, since the patient did not receive the desired number of cycles of chemotherapy, our goal was not achieved, although our hypothesis was sound. 

In summary, a key take-home message from this report is the fact that metastatic malignancy should not be considered an absolute contraindication to ECMO use. As described above, selective use of ECMO can be beneficial in some, but not all, patients with cancer. In our patient, despite the extensive metastatic disease burden in multiple organ systems, factors that motivated us to initiate treatment with VAV-ECMO included the patient’s young age and the conventionally chemo-sensitive nature of the primary tumor. Management of patients with cancer requires a multidisciplinary approach, thus another motivating factor for us was support from our oncology and critical care colleagues in taking ownership of this complex patient. Furthermore, and specifically with regard to using ECMO as a bridge to chemotherapy, the use of VAV-ECMO can be uniquely beneficial in ensuring appropriate distribution of chemotherapy to the lungs. Finally, management of ECMO patients undergoing pharmacologic treatment of a highly sensitive tumor requires close monitoring to ensure appropriate detection and treatment of downstream adverse events (tumor lysis syndrome, renal failure, infection, cytopenias, etc.). 

## Conclusions

Ongoing research and innovation in the ECMO domain have generated increasing evidence supporting its use outside of traditional indications. One area of continued investigation is the use of ECMO in patients with cancer, in which respiratory failure remains a common cause of ICU admission. Recent reports have described the successful use of both veno-venous ECMO and VA-ECMO as a bridge to chemotherapy in patients with malignant neoplasms suffering from cardiopulmonary failure. In select cases, VAV-ECMO holds promise and merits consideration using a multidisciplinary approach. 
